# Enhancing the Physical, Antimicrobial, and Osteo/Odontogenic Properties of a Sol–Gel-Derived Tricalcium Silicate by Graphene Oxide for Vital Pulp Therapies

**DOI:** 10.3390/jfb15070193

**Published:** 2024-07-13

**Authors:** Mohamed Mahmoud Abdalla, Mohammed Zahedul Islam Nizami, Vidhyashree Rajasekar, Mohammed Basabrain, Christie Y. K. Lung, Cynthia Kar Yung Yiu

**Affiliations:** 1 Paediatric Dentistry, Faculty of Dentistry, The University of Hong Kong, Hong Kong, Chinavidhya@connect.hku.hk (V.R.); 2 Dental Biomaterials, Faculty of Dental Medicine, Al-Azhar University, Cairo 11651, Egypt; 3 ADA, Forsyth Institute, Cambridge, MA 02142, USA; 4 Restorative Dental Sciences, Faculty of Dentistry, Umm Al-Qura University, Makkah 24382, Saudi Arabia; mb1sa@connect.hku.hk; 5 Restorative Dental Sciences, Faculty of Dentistry, The University of Hong Kong, Hong Kong, China; cyklung@hku.hk

**Keywords:** sol–gel, tricalcium silicate, graphene oxide, vital pulp therapy, human dental pulp stem cells

## Abstract

Objectives: This study developed a sol–gel tricalcium silicate/graphene oxide (TCS-GO) composite and examined its physicochemical properties, antimicrobial activity, and osteo/odontogenic effect on dental pulp stem cells. Methods: Tricalcium silicate was synthesized and combined with graphene oxide at three different concentrations, namely 0.02%, 0.04%, and 0.08% *w*/*w*, while tricalcium silicate and mineral trioxide aggregate served as controls. The setting time, compressive strength, pH, and calcium ion release of the composites were evaluated, as well as antimicrobial properties against Streptococcus mutans and Lactobacillus acidophilus. Additionally, the viability of dental pulp stem cells; apatite forming ability; and the gene expression of Alkaline phosphatase, Dentin sialophosphoprotein, and Runt-related transcription factor 2 were assessed. Results: TCS-GO (0.08%) showed a significantly shorter setting time and higher compressive strength when compared to MTA (*p* < 0.05). Additionally, tricalcium silicate and TCS-GO groups showed a higher release of Ca ions than MTA, with no significant difference in pH values among the different groups. TCS-GO (0.08%) also demonstrated a significantly stronger antimicrobial effect against Lactobacillus acidophilus compared to MTA (*p* < 0.05). ALP expression was higher in TCS-GO (0.08%) than MTA on days 3 and 7, while DSPP expression was higher in TCS-GO (0.08%) than MTA on day 3 but reversed on day 7. There was no significant difference in RUNX2 expression between TCS-GO (0.08%) and MTA on days 3 and 7. Conclusions: The TCS-GO (0.08%) composite demonstrated superior physicochemical characteristics and antimicrobial properties compared to MTA. Moreover, the early upregulation of ALP and DSPP markers in TCS-GO (0.08%) indicates that it has the potential to promote and enhance the osteo/odontogenic differentiation of DPSCs.

## 1. Introduction

Contemporary approaches to dental treatments emphasize minimally invasive and regenerative techniques [1,2]. A conventional approach to endodontic treatment can weaken teeth and increase the risk of fractures, negatively impacting prognosis and patient satisfaction [1]. Regenerative endodontic procedures (REPs) have emerged as a preferred approach to preserve or restore dental pulp viability [3]. 

Mineral trioxide aggregate (MTA), a calcium silicate (CS)-based material, is considered the gold standard for REPs [4]. However, its use has been negatively affected by poor handling properties, long setting time, contamination with heavy metals, and high cost [5]. Hence, it is imperative to synthesize alternative CS-based biomaterials (mainly di- and tricalcium silicates) to address the limitations of MTA for successful REPs. Ideally, biomaterials used for REPs should exhibit high bioactivity, stimulate odontogenic differentiation of DPSCs, possess sufficient compressive strength to withstand occlusal loads, and demonstrate profound antimicrobial properties against bacterial pathogens that cause pulpal infection and inflammation, impeding the healing process [6,7]. 

The sol–gel preparation of tricalcium silicate (TCS) has been shown to enhance its bioactivity, biocompatibility, and setting time [5,8]. In a previous study, we reported the improved characteristics of sol–gel-derived tricalcium silicate in comparison to MTA [5]. However, it still falls short in terms of sufficient compressive strength, potent antimicrobial effects against cariogenic bacteria, and odontogenic potential [9].

Since its discovery in 2004, graphene, a two-dimensional nanomaterial, has garnered significant attention due to its remarkable mechanical, thermal, and electrical properties [10]. Graphene oxide (GO) is a derivative of the graphene family, consisting of carbon atoms bonded to oxygen. It comprises carboxyl, epoxyl, and hydroxyl functional groups distributed throughout its two-dimensional structure and edges [11]. GO has an extremely large specific surface area with excellent mechanical and physical properties [12]. Studies have shown that GO improved the mechanical strength of Portland cement [13]. Additionally, GO has demonstrated effective antibacterial properties against various oral pathogens [14] and enhanced angiogenic potential [15]. 

It is well known that composite biomaterials are superior to single ones in achieving more desirable properties. By integrating sol–gel-derived TCS with GO, it is possible to enhance the odontogenic potential, antimicrobial activity, and mechanical strength. This promising composite could play a pivotal role in the success and prognosis of REPs.

Some studies have incorporated graphene oxide (GO) into calcium silicate (CaSiO_3_ and dicalcium silicate) and MTA using various methods, reporting a significant increase in the cement’s strength at specific GO concentrations [13,16,17,18,19]. However, no studies to date have investigated the potential impact of GO integration in sol–gel-derived tricalcium silicate (TCS) on the antimicrobial properties against cariogenic biofilms, and the odontogenic potential of the cement for potential use in regenerative endodontic procedures (REPs). It is worth noting that different synthesis methods of GO result in GO with varying properties [20], making it essential to also evaluate the physicochemical and biological properties of the sol–gel-derived TCS-GO composite that utilized a different synthesis method of GO than the ones used in other studies.

Therefore, this study aimed to synthesize and characterize a sol–gel-derived TCS-GO composite; assess its physicochemical, antimicrobial, and odontogenic differentiation properties; and compare it to conventional TCS and commercially available MTA. The null hypothesis tested was that there is no significant difference in physicochemical and antimicrobial properties as well as the biological properties of DPSCs among the TCS-GO composites and the control groups (TCS and MTA).

## 2. Materials and Methods

### 2.1. Synthesis and Characterization of TCS and GO

TCS was synthesized using the sol–gel technique as per our previous work [5]. First, tetraethyl orthosilicate (Si (OC_2_H_5_)_4_; TEOS, Sigma-Aldrich, St. Louis, MO, USA) was mixed with 4 mL deionized water (DIW) and 40 µL of HNO_3_ (69%, VWR PROLABO CHEMICALS, Radnor, PA, USA). Then, Ca (NO_3_)_2_ 4H_2_O (Sigma-Aldrich, St. Louis, MO, USA) was added with continuous mixing for 1 h. The molar ratio of Ca:Si was 3:1. The mix was kept at 60 °C for 24 h and dried at 120 °C for 48 h. The dried gel was sintered at 1400 °C for 4 h. Finally, the calcined powder was ground in a ball mill and sieved. 

GO was synthesized by a modified method adopted according to previous studies [21,22,23]. Briefly, 300 mg of graphite (SP-1, Bay Carbon Inc., Bay City, MI, USA) was added to 75 mL of H_2_SO_4_ (Wako Pure Chemical Industries, Ltd., Osaka, Japan) under magnetic stirring at 200 rpm. A concentration of 900 mg of KMnO_4_ (Wako Pure Chemical Industries, Ltd., Japan) was then gradually dispensed into the mixture. The temperature of the mixture was maintained below 35 °C using an ice bath. Before quenching, this tri-mixture was kept for 2 h under magnetic stirring at 35 °C. Later, the resulting mixture was diluted using 75 mL of ultrapure H_2_O (Milli-Q, Millipore, Merck Ltd., Tokyo, Japan) under vigorous stirring and cooling to control the temperature within or below 50 °C. Finally, the suspension was treated with 7.5 mL of 30% (mass/mass) H_2_O_2_ for 30 min under continuous stirring at ambient temperature. The final product was decontaminated by repeated centrifugation using ultrapure H_2_O. 

#### 2.1.1. Characterization of the Synthesized Materials

The synthesized materials were characterized by an X-ray diffractometer (XRD, Rigaku SmartLab 9 kW, Tokyo, Japan) supplied with a copper rotating anode (K 1 1.541, Ka2 1.544) at 200 mA current and 45 kV voltage. Diffraction peaks were matched using JADE pattern identification software (JADE v. 6.5, San Jose, CA, USA). Further, the samples were also characterized using attenuated total reflection/Fourier-transformed infrared (ATR-FTIR, L160000 Spectrum-Two spectrophotometer instrument; PerkinElmer Inc., Shelton, CT, USA) at 400–4000 cm^−1^ wavenumber.

#### 2.1.2. Mixing of TCS-GO Composites

The GO stock (10 mg/mL) was dispersed in deionized water and used as the liquid phase in the cement formulation. The concentration of GO within the liquid phase was adjusted to achieve three final concentrations of TCS-GO (0.02%, 0.04%, and 0.08%) *w*/*w*. The powder-to-liquid ratio was 2:1. TCS and MTA (Dentsply, Tulsa Dental, OK, USA) served as control groups.

### 2.2. Setting Time and Compressive Strength

The setting time assessment followed ASTM C266-15, using the Gilmore needle indentation technique with 5 disc-shaped specimens, each with a diameter of 10 ± 0.1 mm and a thickness of 2 ± 0.1 mm. The Gilmore needle (diameter: 2 ± 0.1 mm and weight: 453.5 ± 5 g) was applied to the sample surface at 180 s intervals, continuing until no noticeable indentations were observed.

For the compressive strength evaluation, the ISO 9917-1 protocol was followed. Six cylindrical specimens per group (diameter = 3 mm, height = 6 mm) were produced and subjected to compressive strength testing after a 7-day incubation period at 37 °C and 95 ± 5% humidity. An ElectroPuls™ E3000 universal testing machine (Instron, Norwood, MA, USA) was employed to determine the compressive strength at a crosshead speed of 1.0 mm/min until sample fracture occurred. The compressive strength was calculated in megapascals (MPa) using the following formula:

σ = 4F/πd2

where σ represents the compressive strength (MPa), F is the maximum load before failure, and d is the specimen’s diameter in mm.

### 2.3. pH Measurements

Three-disc specimens per group were prepared (diameter of 10 ± 0.1 mm and a height of 2 ± 0.5 mm) and submerged in a plastic tube containing 15 mL of Hank’s Balanced Salt Solution (HBSS) [24]. A calibrated pH meter was employed to measure the pH after 3 h, 24 h, and 72 h of immersion in HBSS, with HBSS alone serving as the control solution. The pH meter was calibrated prior to each test.

### 2.4. Calcium Ion Release

The calcium ion release was measured using the ICP-OES after 1, 3, and 7 days of immersing the samples in DIW. The collected solutions were then diluted and inserted into an ICP-OES machine to detect the calcium ion concentration against the plotted standard curve.

### 2.5. Antimicrobial Test

*Streptococcus mutans* (*S. mutans*, UA159) (ATCC 700610) and *Lactobacillus acidophilus* (*L. acidophilus*) (ATCC 9224) were grown in brain heart infusion (BHI) broth at 37 °C under anaerobic conditions (85% N_2_, 10% H_2_, 5% CO_2_). The bacterial suspension concentration was adjusted to 10^7^ CFU/mL using the McFarland spectrophotometric method at OD600 nm.

#### Colony Forming Units (CFUs)

The prepared discs of different experimental composites were placed in 12-well plates and incubated in 1 mL of 1:1 bacterial suspension (10^7^ CFU/mL) for 24 h under anaerobic conditions for biofilm formation. The discs were then suspended and vortexed in 1 mL PBS for 1 min. Then, 50 μL of the detached biofilms was spiral-plated on agar plates at different dilutions and incubated anaerobically for 48 h. The bacterial colonies were counted, and CFU was estimated according to the following formula: Bacteria CFU per mL = Average number of colonies for a dilution × 50 × dilution factor. Bacterial CFU was expressed as the mean ± standard deviation log10 CFU/mL.

### 2.6. Viability of DPSCs

#### 2.6.1. DPSCs’ Isolation and Characteristics

The protocol for isolating DPSCs was approved by the Institutional Review Board, the University of Hong Kong/Hospital Authority Hong Kong West Cluster (UW 22-542). After collecting the informed consent from the patient, DPSCs were extracted from freshly removed caries-free impacted third molars belonging to 27–32-year-old male/female patients. The extraction was performed at the Oral and Maxillofacial Clinic, Prince Philip Dental Hospital. After tooth extraction, under aseptic conditions, the extracted molar was transferred immediately to the cell culture hood in alpha-minimum essential medium (α-MEM). The dental follicle and pulp tissues were promptly separated, and DPSCs were isolated using the technique outlined by Suchanek et al. [25]. The DPSCs were then cultivated in α-MEM enhanced with 10% (*v*/*v*) fetal bovine serum (FBS) and 1% (*v*/*v*) penicillin–streptomycin antibiotic solution (ThermoFisher Scientific, Inc., Waltham, MA, USA) at 37 °C in a 5% CO_2_ incubator. The medium was refreshed every other day. Finally, DPSCs were incubated with antibodies against CD45, CD73, CD90, and CD105 (Abcam, Cambridge, UK) followed by a fluorescence-activated cell sorter (FACS) for characterization and assessment of their stemness.

#### 2.6.2. CCK-8 Assay

The viability of DPSCs was evaluated against different control and experimental groups using the CCK-8 assay kit. Briefly, DPSCs were cultured in α-minimal essential medium (α -MEM, GIBCO BRL, Gaithersburg, MD, USA) supplemented with 10% fetal bovine serum, penicillin, and streptomycin at 37 °C, 5% CO_2_. The experimental materials were first mixed and prepared within the individual wells of a 96-well plate. Following the setting of the material, cells were then seeded directly onto the surface of the set materials in each well with a density of 10,000 cells/well for 24 h at 37 °C, 5% CO_2_. They were then treated with experimental materials. After 24 h, the treated cells were assessed for cell viability using a CCK-8 assay kit (Sigma-Aldrich, St. Louis, MI, USA) following the manufacturer’s recommendations. The optical density (OD) was read at an end-point absorbance of 450 nm in a microplate reader (Spectra max m2, Molecular devises, Sunnyvale, CA, USA). Data were presented in % viability after normalization to the untreated control group.

#### 2.6.3. Live and Dead Staining Assay

The cell viability was further assessed using the LIVE/DEAD kit (Cat. No. L3224, Gibco-Invitrogen, Carlsbad, CA, USA) according to the manufacturer’s instructions. DPSCs were seeded in a 48-well plate at a density of 3 × 10^4^ cells/well for 24 h at 37 °C, 5% CO_2_, and then exposed to experimental materials for 24 h. The cells were washed with PBS and stained with the live and dead stain for 1 h followed by imaging the cells using a fluorescence microscope (Leica DMi8, Leica Microsystems Ltd., Durham, NC, USA).

### 2.7. In vitro Apatite Forming Ability

The in vitro apatite formation of the fabricated composites was evaluated by soaking them in phosphate-buffered saline (PBS) [26]. Disc-like specimens (5 mm in diameter and 2 mm thickness) were prepared and immersed in PBS for 1 and 7 days. At the end of each immersion time point, the discs were gently rinsed with DIW water. Finally, the Ca-to-P ratio was estimated using an energy dispersive X-ray (EDX, 1XRF System, Austin, TX, USA) analyzer at an accelerating voltage of 15 kV, and then under SEM (SU1510, Hitachi, Ibaraki, Japan) after sputter-coating to assess the formed apatite-like crystals on their surface.

### 2.8. Quantitative Real-Time PCR (RT-qPCR)

DPSCs were cultured in 6-well plates at a density of 2 × 10^5^ cells/well for 24 h. The seeded cells were then incubated with the TCS/GO treatment groups for 3 and 7 days. The total RNA of the treated DPSCs was extracted using an RNeasy extraction kit (Qiagen, Hilden, Germany). The RNA concentration was quantified using a spectrophotometer (NanoDrop. 2000, Thermo Fisher Scientific, Waltham, MA, USA) and reverse-transcribed to complementary DNA (cDNA) using SuperScript Vilo Mastermix (Invitrogen, Carlsbad, CA, USA). Quantitative real-time polymerase chain reaction was performed using the ABI Prism 7000 Sequence Detection System (Applied Biosystems, Carlsbad, CA, USA) with Takara SYBR green. Reactions were performed at 95 °C for 10 min followed by 40 cycles of 95 °C for 15 s and 60 °C for 1 min. In terms of primers for the HDPCSs, Alkaline phosphatase (ALP), Dentin sialophosphoprotein (DSPP), Runt-related transcription factor 2 (RUNX-2), and the housekeeping gene glyceraldehyde-3-phosphate dehydrogenase (GAPDH) were used. The primer sequences used in PCR are shown in Table 1. For data analysis, the StepOne software v2.0.2 (Applied Biosystems) calculated the levels of target gene expression in samples relative to the level of expression in the control samples with the comparative cycle threshold method (ΔΔCT). The 2^−∆∆Ct^ method was used to analyze relative changes in gene expression.

#### Statistical Analysis

Data were subjected to statistical analysis using SPSS v. 27. (IBM Statistics, New York, NY, USA). The setting time, compressive strength, CFU, and RT-qPCR quantitative variables were analyzed using one-way ANOVA with the Bonferroni post hoc test. Calcium ion release and pH measurements were analyzed using two-way ANOVA (factor 1: groups, factor 2: time points) with the Bonferroni post hoc test. The non-parametric cell viability data were analyzed using the Kruskal–Wallis H test with Dunn’s correction for post hoc comparison. The level of significance was set at (*p* < 0.05).

## 3. Results

### 3.1. Dental Pulp Stem Cell Isolation

**Figure 1:**
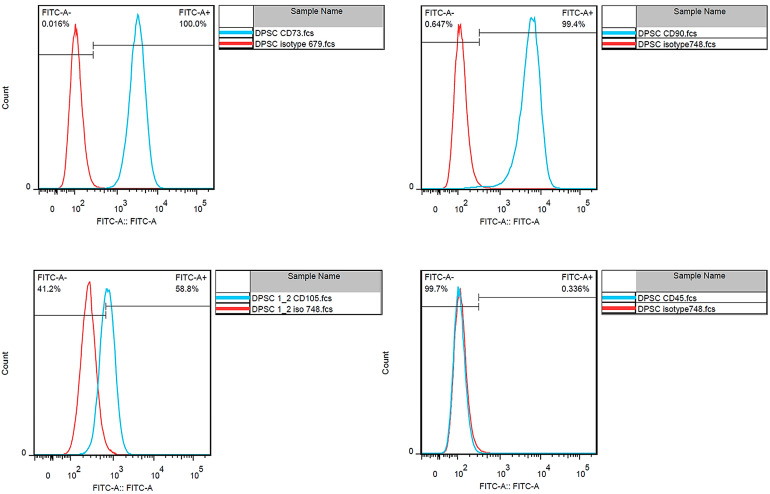
The flow cytometry results showed characteristic markers of DPSCs CD73 (100%), CD90 (99.4%), and CD105 (58.8%) and lacked an expression of CD45 (0.336%).

The flow cytometry analysis revealed that the isolated cells expressed CD73 (100%), CD90 (99.4%), and CD105 (58.8%) and lacked an expression of CD45 (0.336%), which represents typical mesenchymal stem cell markers (Figure 1). 

### 3.2. Characterization of the Prepared Materials

**Figure 2:**
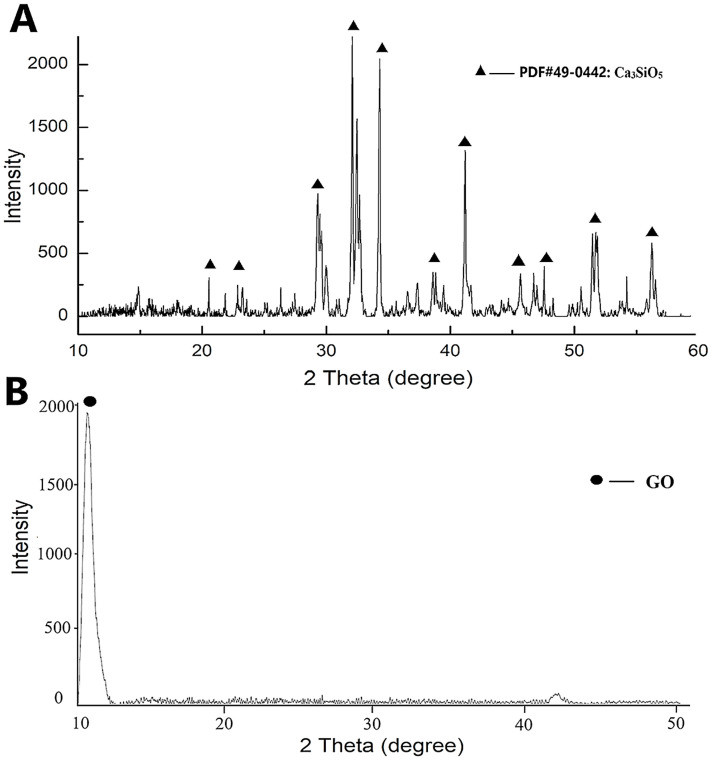
X-ray diffraction (XRD) analysis revealed the distinctive peaks associated with tricalcium silicate (Ca_3_SiO_5_) (**A**) and graphene oxide (GO) (**B**).

**Figure 3:**
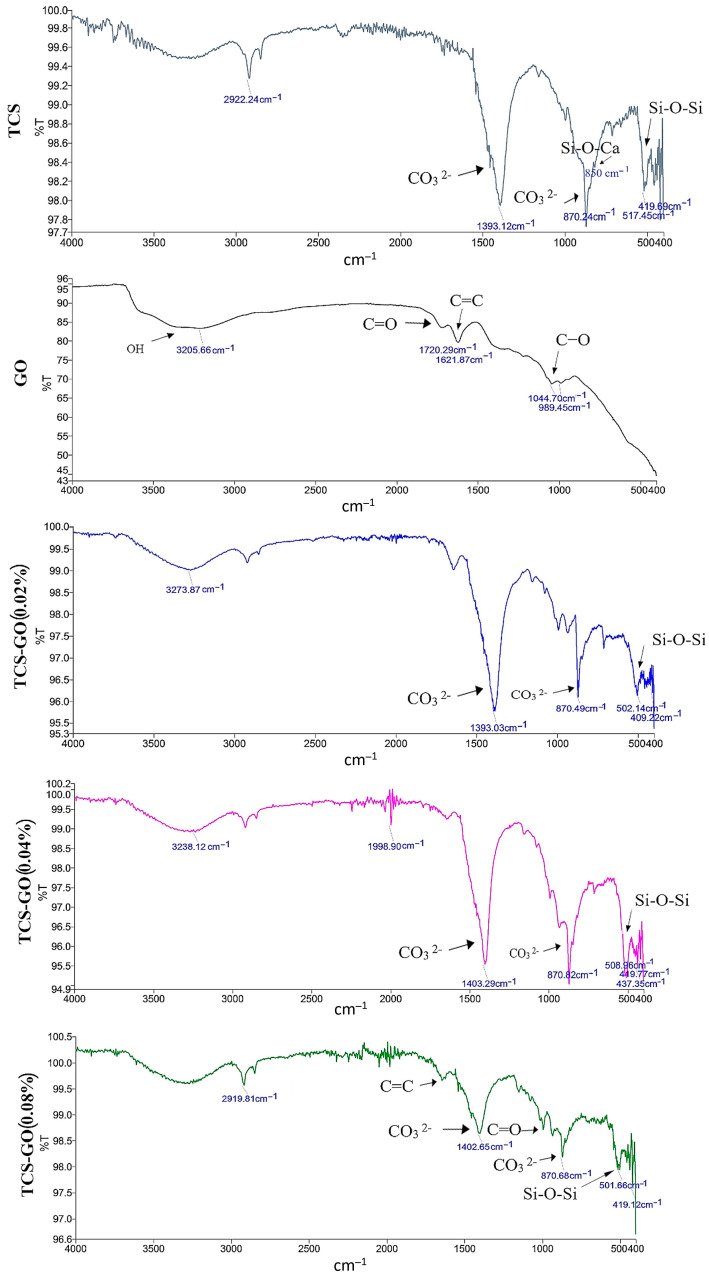
FTIR analysis of TCS-GO composites revealed characteristic peaks for O-Si-O, Si-O-Ca, and calcite’s carbonate group in TCS, as well as C-O, C=C, C=O, and OH groups in GO. These peaks were observed in various TCS-GO composites with different GO weight percentages.

The phase compositions of the synthesized TCS and GO are shown in Figure 2. Data presented typical diffraction peaks of TCS (Ca_3_SiO_5_) at 29.2°, 33.1°, 34.2, 41.6° 2θ. While for GO, an intense diffraction peak at 11.5° 2θ was identified [27]. The FTIR spectra of the set cements of TCS and TCS-GO composites are shown in Figure 3. For the TCS spectrum, the adsorption peaks at 419 cm^−1^ and 517 cm^−1^ are due to the bending vibrations of O-Si-O. The adsorption peak at 850 cm^−1^ is due to the vibration of Si-O-Ca in TCS. The adsorption peaks at 870 cm^−1^ and 1393 cm^−1^ are due to the carbonate group (CO_3_^2−^) of calcite [28]. For the GO spectrum, the adsorption peaks at 989 cm^−1^ and 1044 cm^−1^ are due to the vibration of C-O. The adsorption peak at 1621 cm^−1^ is due to the vibration of C=C. The adsorption peak at 1720 cm^−1^ is due to the vibration of the carbonyl group, C=O. The adsorption peak at 3205 cm^−1^ is due to the OH group [29]. The adsorption peaks at 409 cm^−1^ and around 508 cm^−1^, which are due to the bending vibrations of O-Si-O in TCS, are observed for the three composites. The adsorption peaks at 870 cm^−1^ and around 1400 cm^−1^ in TCS, which are due to the carbonate group (CO_3_^2−^) of calcite, are observed. There was no change in the structure of TCS after the incorporation of GO.

### 3.3. Setting Time and Compressive Strength

**Figure 4:**
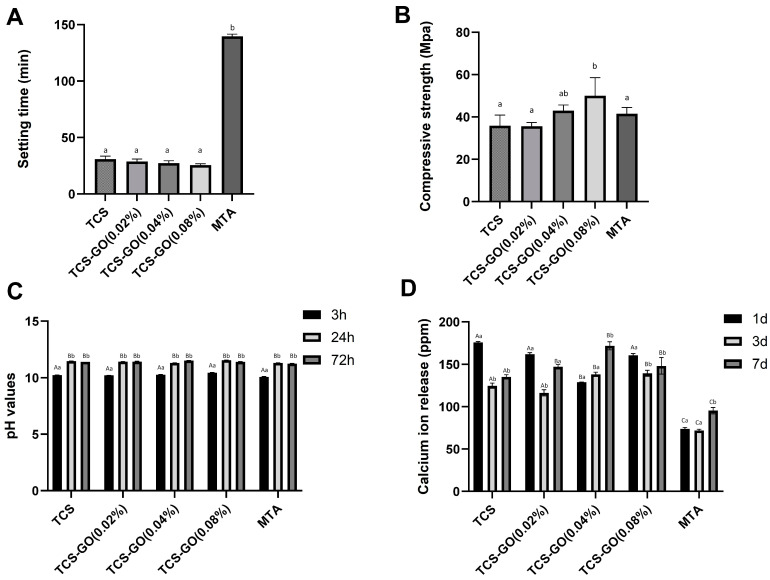
(**A**) The setting time of the TCS-GO and control groups. (**B**) The compressive strength of the TCS-GO and control groups. Different lowercase letters indicate significant differences between the groups as indicated by one-way ANOVA with Bonferroni post hoc test (*p* < 0.05). (**C**) The pH values of the prepared TCS-GO composites. (**D**) Calcium ion release in ppm of the prepared cements. Different lowercase letters represent significant differences between the groups, while different uppercase letters show significant differences within individual groups as indicated by two-way ANOVA (factor 1: groups, factor 2: time points) with Bonferroni post hoc test (*p* < 0.05).

The final setting times of TCS and TCS-GO composites were significantly shorter than MTA (*p* < 0.001), while no significant difference in setting time was found between TCS and TCS-GO (0.02%), TCS-GO (0.04%), and TCS-GO (0.08%) composites (Figure 4A).

The compressive strength of TCS-GO (0.08%) composite was significantly higher than TCS, TCS-GO (0.02%), and MTA (*p* < 0.05). No significant difference was found between TCS, TCS-GO (0.02%), TCS-GO (0.04%), and MTA (*p* > 0.05). The TCS/GO (0.08%) composite showed a 45% increase in compressive strength compared to conventional TCS and a 25% increase in compressive strength compared to MTA (Figure 4B). 

### 3.4. pH Measurements

The results of two-way ANOVA showed that factor 1 (groups) was statistically non-significant (*p* > 0.05), and factor 2 (time) was statistically significant (*p* < 0.05), while their interactions were statistically non-significant (*p* > 0.05). The pH value of TCS, TCS-GO, and MTA increased with time from 3 h to 24 h and remained stable at 72 h. There was no significant difference in pH values among the tested and control groups (*p* > 0.05) (Figure 4C). 

### 3.5. Calcium Ion Release

The results of two-way ANOVA showed that factor 1 (groups) and factor 2 (time), as well as their interactions, were statistically significant (*p* < 0.05). TCS and TCS-GO composites displayed a significant average increase of 46% in Ca release compared to MTA at all time points (*p* < 0.05) (Figure 4D).

### 3.6. Colony Forming Units

**Figure 5:**
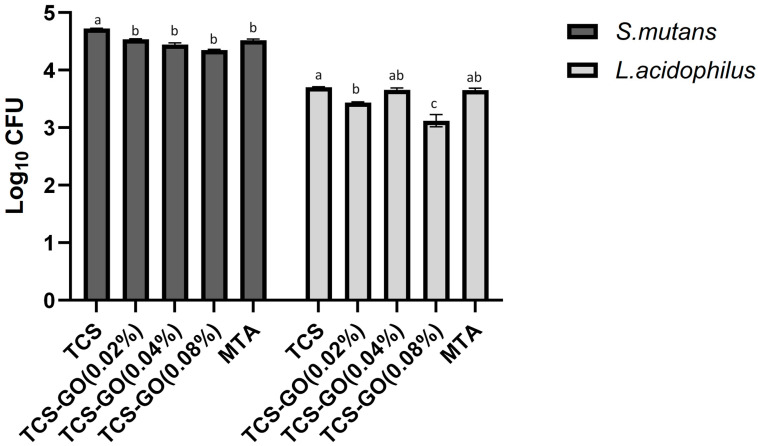
Colony forming units of *S. mutans* and *L. acidophilus*. Different lowercase letters indicate significant differences between the groups at each bacterial strain, as indicated by one-way ANOVA with Bonferroni post hoc test (*p* < 0.05).

The results of the CFU assay are presented in Figure 5. A comparison of viable *S. mutans* colonies showed that all the TCS-GO composite groups and MTA exhibited a significantly lower CFU/mL count compared to the TCS group (*p* < 0.05). However, no significant differences in CFU/mL counts were observed among the TCS-GO composite groups and MTA (*p* > 0.05). Regarding *L. acidophilus*, TCS-GO (0.08%) exhibited a significantly lower CFU/mL count compared to all other groups (*p* < 0.05). There were no significant differences in CFU/mL counts among the control, TCS-GO (0.04%), and MTA groups (*p* > 0.05).

### 3.7. Cell Viability (CCK-8 Assay)

**Figure 6:**
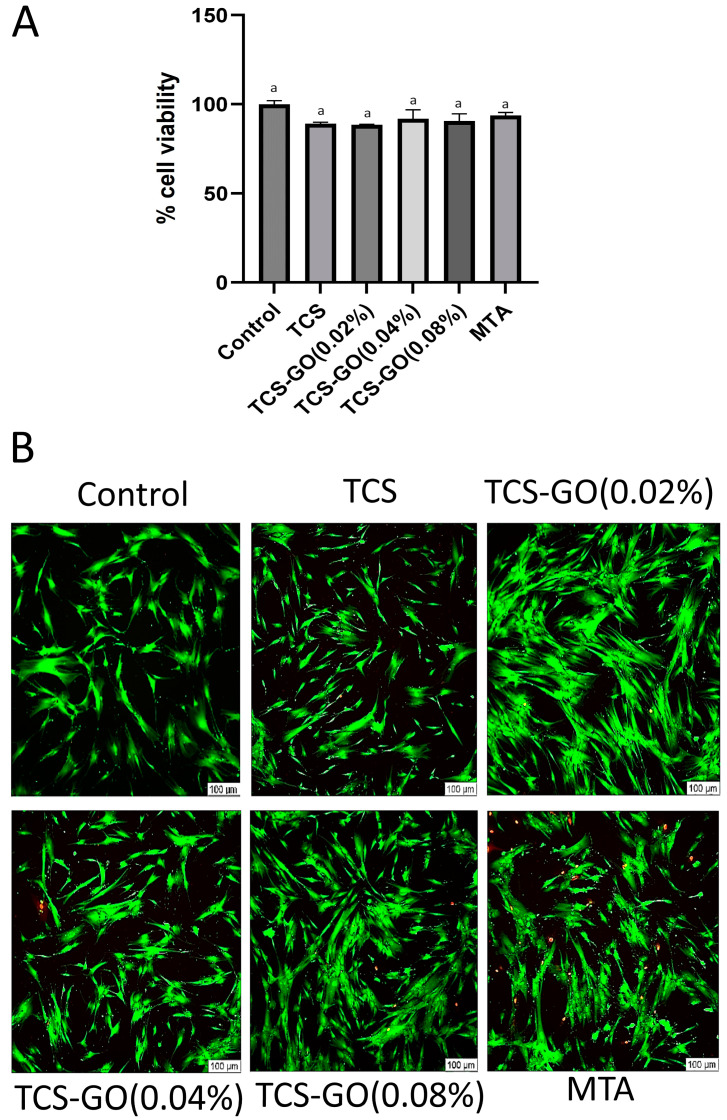
(**A**) The DPSC viability 10^4^ cell/well represented as percent viability compared to the control group (100%) viability. Different lower case letters indicate significant difference (*p* > 0.05). (**B**) The live and dead staining images display that the majority of the cells are live (indicated by green), while only a small proportion of the cells are dead (indicated by red).

Both TCS and TCS-GO composites were biocompatible and expressed over 88% cell viability. No significant difference in cell viability was found between the experimental groups and control and MTA groups (*p* > 0.05) (Figure 6A).

#### Live and Dead Staining Assay

Live and dead images revealed predominantly live cells (indicated by green staining) were observed in all treatment and control groups, with no discernible differences among them, except for MTA which displayed some dead cells. A minimal number of dead cells (marked by red staining) were detected among the other groups (Figure 6B). 

### 3.8. In Vitro Apatite Forming Ability

**Figure 7:**
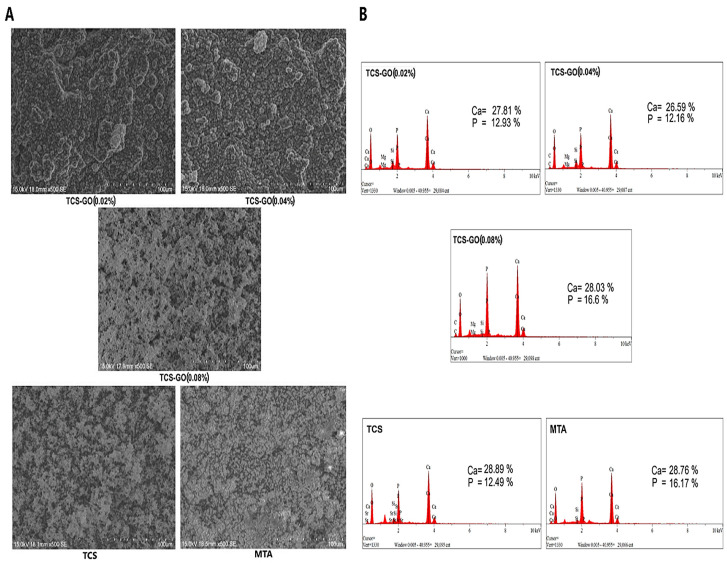
SEM images illustrate the formation of an apatite-like layer on the examined cements (**A**). EDX analysis of the fabricated cements with the Ca/P ratio (**B**).

The formation of an apatite-like layer on the surfaces of samples was assessed using SEM and EDX elemental analysis. A well-developed apatite layer was formed on the surfaces of all experimental TCS-GO composites, which was comparable to TCS and MTA. A slight increase in the apatite layer deposition was seen in the TCS-GO (0.08%) group compared to the TCS-GO (0.02%) and TCS-GO (0.04%) groups (Figure 7A). EDX elemental analysis displayed a marked increase in the atomic % of P in all groups after 7 days of immersion in PBS, which indicates the possible formation of hydroxyapatite-like crystals (Figure 7B).

### 3.9. Quantitative Real-Time PCR (RT-qPCR)

**Figure 8:**
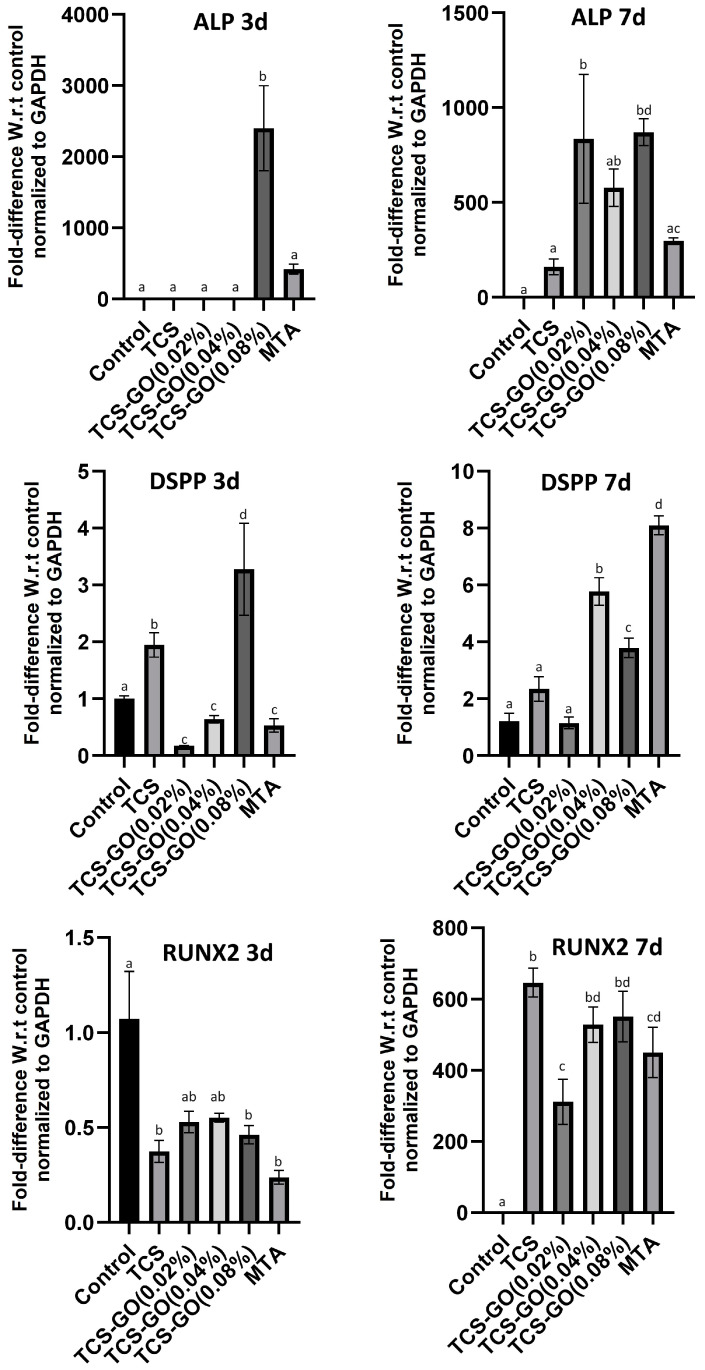
Relative gene expression of ALP, DSPP, and RUNX2 at 3 and 7 days. Different lower-case letters represent significant differences between groups as indicated by one-way ANOVA with Bonferroni post hoc test (*p* < 0.05).

The relative gene expression of ALP, DSPP, and RUNX2 are presented in Figure 8. ALP expression was significantly higher in the TCS-GO (0.08%) group on day 3 compared to other groups (*p* < 0.05), and the level of ALP expression remained higher than TCS and MTA groups on day 7. DSPP expression in the TCS-GO (0.08%) group significantly increased on day 3 compared to all other groups (*p* < 0.05). On day 7, DSPP expression in the TCS-GO (0.08%) group remained higher than TCS, TCS-GO (0.02%) groups but became lower than TCS-GO (0.04%) and MTA (*p* < 0.05) groups. There was no significant difference in RUNX2 expression among TCS-GO (0.04%), TCS-GO (0.08%), and MTA groups on days 3 and 7.

## 4. Discussion

The results of the present study showed that the TCS-GO (0.08%) composite exhibited significant improvements in setting time and compressive strength when compared to MTA. Additionally, the TCS-GO (0.08%) composite demonstrated greater antimicrobial properties against *L. acidophilus* and enhanced expression of the osteo/odontogenic genes in DPSCs. Based on these results, it can be concluded that the TCS-GO (0.08%) composite has the potential to replace MTA in REPs after validating the findings with an in vivo animal model; thus, the null hypothesis tested has to be rejected.

Graphene oxide is one of the promising materials, it has been incorporated into different materials to improve their biological and physical properties [11,17]. GO has been effectively incorporated into mineral trioxide aggregate (MTA) and other orthopedic materials, enhancing their performance [18,19]. This has led to an improved biological response, providing better cell adhesion and proliferation for bone tissue engineering applications. 

The sol–gel synthesis method was chosen for the development of pure TCS due to its established effectiveness in producing TCS with superior properties compared to other commonly used synthesis methods for MTA [5,30,31,32]. Since sol–gel synthesized TCS has proven to have better properties than MTA [5], we proposed incorporating GO into TCS than into MTA, as performed in previous studies [19]. The TCS-GO composites exhibited significantly shorter setting times compared to MTA. This can potentially reduce treatment time and improve patient comfort during dental procedures [33]. The sol–gel process resulted in the formation of small particle sizes and a porous structure, which likely provided a larger surface area for the chemical setting reaction. This, in turn, facilitated rapid dissolution and recrystallization during the hydration process [34]. 

The presence of GO has been shown to affect the hydration process of calcium silicate-based materials [35]. However, the minimal effect of GO on the setting time of TCS-GO composites in our study may be attributed to the low concentrations of GO used. Dubey et al. [17] examined the effects of incorporating GO nanosheets into calcium silicate-based bioactive cements and discovered that adding 3 wt.% GO nanosheets considerably reduced the setting time of the tested cements. Our findings contrast with those of Dubey et al., potentially corroborating our earlier explanation that the low GO concentrations utilized in our study (0.02, 0.04, and 0.08 wt.%) did not have a significant impact on shortening the setting time of TCS-GO composites. 

Another important finding is the enhanced compressive strength of TCS-GO (0.04%) and TCS-GO (0.08%) composites compared to TCS and MTA. The increased compressive strength improves the material’s ability to withstand mechanical stresses during mastication and other functional activities. This improvement in compressive strength can be attributed to the incorporation of GO, which is known for its excellent mechanical properties and ability to reinforce composite materials [36]. Our findings align with other studies that have incorporated GO at low concentrations into calcium silicate-based cements and observed a significant increase in compressive strength [37,38,39]. 

TCS and TCS-GO composites exhibited higher calcium ion release than MTA at all time points, which contributes significantly to the remineralization process [40]. Moreover, we did not observe any significant drop in the pH of the TCS-GO composite, despite the acidic pH of the synthesized GO dispersion. It is expected that the high alkalinity of TCS could have a buffering effect on the acidity of GO. The biocompatibility and cell viability of TCS and TCS-GO composites were comparable to those of TCS and MTA. Several studies have explored the biocompatibility of TCS, which has shown high cell viability with negligible adverse effects on cells [34,41]. Furthermore, the cytotoxicity of GO was evaluated in a previous study, which did not exhibit any cytotoxic effect on cultured cells [22], consistent with our findings. 

The apatite formation capabilities of TCS-GO composites were similar to those of TCS and MTA, indicating their potential to support the mineralization process and bond with the tooth structure. The TCS-GO (0.08%) group demonstrated slightly higher apatite layer deposition, which may further enhance the material’s performance in VPT. Shie et al. [15] evaluated the bioactivity of calcium silicate–graphene oxide composite after soaking in simulated body fluids (SBFs) for 1 h and 24 h and reported a uniform formation of an apatite-like layer on the tested samples, which is consistent with our findings. 

Moreover, we investigated the antimicrobial properties of TCS-GO composites. The presence of cariogenic bacteria such as *S. mutans* and *L. acidophilus* in the vicinity of the exposed pulp can have a detrimental effect on the success of REPs as they contribute to persistent inflammation in pulp tissues [42]. These bacteria can invade the dentinal tubules and release toxins, leading to the inflammation and destruction of the pulp tissue. The antimicrobial activity of TCS-GO composites was significantly better than the TCS group, with TCS-GO (0.08%) showing the most promising results. When GO forms a composite with another substance, it can enhance the antimicrobial activity of the original material [22,43]. This improvement in antimicrobial properties is crucial for preventing bacterial infections and promoting healing in REPs. 

The findings of our study suggest that the TCS-GO composite has a positive effect on the differentiation of DPSCs into osteogenic and odontogenic lineages. The upregulation of ALP and DSPP expression in TCS-GO (0.04%) and TCS-GO (0.08%) groups at 3 and 7 days indicates the capability of TCS-GO composite to promote DPSCs differentiation. Alkaline phosphatase (ALP) is involved in mineralization and is considered a marker for early osteogenic and odontogenic differentiation [44]. Dentin sialophosphoprotein (DSPP) is a marker specific to odontogenic differentiation, as it is involved in dentin formation and mineralization. The upregulation of DSPP expression in the TCS-GO (0.08%) group on day 3 implies that the TCS-GO composite may support DPSCs’ differentiation into odontoblast-like cells and promote dentin formation. An in vitro study examined the impact of graphene on the osteogenic and odontogenic differentiation of DPSCs, revealing that graphene can promote the osteogenic differentiation of DPSCs but not odontogenic differentiation [45]. However, our results suggest the odontogenic differentiation of DPSCs, which could be attributed to the distinct GO utilized in this study and its composite formulation with CS. It is worth noting that the bioactive properties of graphene oxide may vary depending on the specific graphene products and synthesis methods used, similar to the differences in cytotoxicity observed among different graphene materials [20]. Runt-related transcription factor 2 (RUNX2) is a critical transcription factor for both osteogenic and odontogenic differentiation, as it regulates the expression of genes involved in bone and dentin formation. The RUNX2 expression was downregulated at 3 days, while at 7 days, all the groups expressed a similar increase in the gene expression compared to 3 days. 

While the present study shows promising results, it is important to acknowledge some limitations. The experiments were conducted in vitro, which may not fully replicate the complex environment of the oral cavity and dental pulp tissue in vivo. Future studies should include in vivo animal models to better understand the material’s behavior and effectiveness in a more realistic setting. Secondly, our study focused on the short-term effects of the TCS-GO composite on DPSCs’ differentiation and antimicrobial properties, while the long-term effects should be further investigated. 

## 5. Conclusions

TCS-GO (0.08%) had a shorter setting time, higher compressive strength, calcium release, and antimicrobial properties compared to MTA materials. The DPSC viability did not express any significant reduction after the incorporation of GO, indicating the cytocompatibility of the tested materials. The upregulation of ALP and DSPP markers also indicated the potential of the composite to enhance the osteo/odontogenic differentiation of DPSCs. These findings suggest that TCS-GO could be a promising option for regenerative endodontic procedures.

## Figures and Tables

**Table 1:** Primer sequences used for RT-qPCR.

	Primers
*DSPP*	F: ATATTGAGGGCTGGAATGGGGA R: TTTGTGGCTCCAGCATTGTCA
*RUNX2*	F: ACTCTACCACCCCGCTGTC R: CAGAGGTGGCAGTGTCATCA
*ALP*	F: ACGTGGCTAAGAATGTCATC R: CTGGTAGGCGATGTCCTTA
*GAPDH*	F: TGGCACCCAGCACAATGAA R: CTAAGTCATAGTCCGCCTAGAAGCA

## Data Availability

The data presented in the study are available upon request from the corresponding author.
